# Characterization and Lifetime Dietary Risk Assessment of Eighteen Pyrrolizidine Alkaloids and Pyrrolizidine Alkaloid N-Oxides in New Zealand Honey

**DOI:** 10.3390/toxins13120843

**Published:** 2021-11-26

**Authors:** Andrew J. Pearson, Jeane E. F. Nicolas, Jane E. Lancaster, C. Wymond Symes

**Affiliations:** 1 New Zealand Food Safety, Ministry for Primary Industries, Wellington 6011, New Zealand; apearson@tonkintaylor.co.nz; 2 Catalyst Ltd., Katikati 3129, New Zealand; jane.lancaster@catalystnz.co.nz (J.E.L.); wymond.symes@catalystnz.co.nz (C.W.S.)

**Keywords:** probabilistic model, plant toxins, lycopsamine, echimidine, retrorsine, senecionine, boraginaceae, asteraceae, honeybee

## Abstract

Pyrrolizidine alkaloids (PAs) are a large group of botanical toxins of concern, as they are considered genotoxic carcinogens, with long-term dietary exposure presenting an elevated risk of liver cancer. PAs can contaminate honey through honeybees visiting the flowers of PA-containing plant species. A program of monitoring New Zealand honey has been undertaken over several years to build a comprehensive dataset on the concentration, regional and seasonal distribution, and botanical origin of 18 PAs and PA N-oxides. A bespoke probabilistic exposure model has then been used to assess the averaged lifetime dietary risk to honey consumers, with exposures at each percentile of the model characterized for risk using a margin of exposure from the Joint World Health Organization and United Nations Food and Agriculture Organization Expert Committee on Food Additives (JECFA) Benchmark Dose. Survey findings identify the typical PA types for New Zealand honey as lycopsamine, echimidine, retrorsine and senecionine. Regional and seasonal variation is evident in the types and levels of total PAs, linked to the ranges and flowering times of certain plants. Over a lifetime basis, the average exposure an individual will receive through honey consumption is considered within tolerable levels, although there are uncertainties over high and brand-loyal consumers, and other dietary contributors. An average lifetime risk to the general population from PAs in honey is not expected. However, given the uncertainties in the assessment, risk management approaches to limit or reduce exposures through honey are still of value.

## 1. Introduction

**Figure 1:**
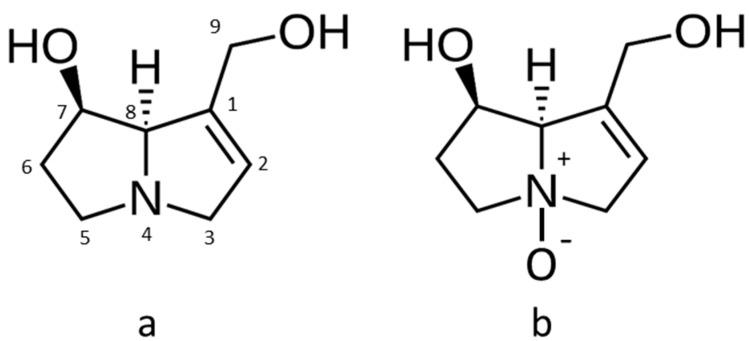
(**a**) A basic pyrrolizidine alkaloid structural skeleton (retronecine) with positions numbered. Congeners differ through substitution of the two hydroxyl groups with more complex functional groups. (**b**) Pyrrolizidine alkaloid N-oxide structural skeleton (retronecine-N-oxide).

Pyrrolizidine alkaloids (PAs) are botanical toxins estimated to be present in over 6000 plants [1]. To date, over 660 different PAs and their corresponding N-oxide derivatives have been characterized. The main plant sources are the families Boraginaceae (all genera), Asteraceae (tribes *Senecioneae* and *Eupatorieae*), and Fabaceae (genus *Crotalaria*) [2]. Different plant species in these families produce characteristic mixtures of 1,2-unsaturated PAs and their saturated analogues, and varying amounts of their corresponding N-oxides. The basic PA structural skeleton is presented in Figure 1. An overview of the structural formula of key PAs and N-oxides has been compiled in the recent Joint FAO/WHO Expert Committee on Food Additives (JECFA) safety evaluation [3]. 

The most common toxic effect in both short-term and long-term studies of 1,2-unsaturated PAs is hepatotoxicity, specifically the epithelial cells of the sinusoids [3]. Human poisoning with 1,2-unsaturated PAs is known to cause liver toxicity resulting in the development of hepatic sinusoidal obstruction syndrome [3]. 

Toxicity from 1,2-unsaturated PAs is a consequence of CYP450 activation through oxidation to the pyrrolic ester metabolites 6,7-dihydro-7-hydroxy-1-hydroxymethyl-5H-pyrrolizine ester (DHP esters or Dehydro-PAs) [3,4]. DHP-esters may be then further hydrolyzed to DHP. As there are several reactive sites in the pyrrolic ester molecule, at the C5/C7 and C9 positions, crosslinking can occur between two sites in DNA or between DNA and protein, hence, PAs are considered mutagenic [2]. Carcinogenicity is considered to be the most critical end-point following long-term exposure of experimental animals to certain PAs. Though the main carcinogenic target organ for PAs is the liver, tumors have also been reported in other tissues, including lung and kidney [3]. PA-N-oxides upon ingestion are reduced in the gastrointestinal tract to the corresponding parent 1,2 unsaturated PAs, presenting the same toxicity when present in the diet [3].

Humans are exposed to PAs and PA-N-oxides through the intake of PA-containing plants and/or PA-contaminated foodstuffs [2,3,5]. There is currently no international Codex Alimentarius standard for the maximum allowable level of PAs in foods. However, work to prepare a discussion paper on potential risk management in food commenced in 2021 [6]. In Europe, maximum levels for PAs in different teas and herbal products come into effect in July 2022 [7].

A prohibition on the sale or use in food of certain named PA-containing plants (for example: *Symphytum officinale*-Comfrey) applies in Australia and New Zealand [8], whereas other countries advise consumers and food business not to use or to ingest them [9]. 

Plant pollens and floral nectar are known sources of PAs, and, hence, their collection by honeybees (*Apis mellifera)* can lead to incorporation into honey and other bee products, such as bee bread and supplements based on pollen, propolis, and royal jelly [2,3,10,11,12,13]. With a foraging range of several kilometers possible for bees from the hive, the growth of PA-containing plants near apiary sites can present an ongoing source of contamination of honey, and a potential dietary exposure risk [14]. Overseas studies have shown the presence of PA concentrations in a high percentage of retail honey, with typically 80–95% of analyzed honey samples showing reported values for individual PAs, up to concentrations of 5600 µg/kg [2,3,10]. The consequence is that honey has the potential to be a notable contributor to overall dietary exposure of PAs [3]. Despite this, general guidance on managing the contamination risk for honey is limited when compared with that for pasture and crop management of PA-containing plants [15]. In the recent JECFA safety evaluation, a noted limitation was that dietary exposure assessments were only available for a small number of countries, and often those studies for honey focused on specific monofloral varieties, and were less relevant to overall honey consumption [3]. 

To date there has been limited analysis of New Zealand honey for PA concentrations. Analysis of nine honey samples, including five monofloral *Echium vulgare* (Vipers Bugloss) honeys for a PA suite focused on *Echium* spp. as a floral source, and reported two samples as having not detected results, whereas the other seven samples had quantified levels of 17–2850 µg/kg total PAs and their N-oxides [16]. Echimidine and echivulgarine were the predominant PAs quantified. These findings were confirmed by a second study that sampled eight New Zealand monofloral *Echium vulgare* honeys and reported a range of 311–411 µg/kg total PAs [17]. In this case, the PA analyte suite considered the other major alkaloids, such as lycopsamine, monocrotaline, and retrorsine, and, again, the conclusion was that echimidine was the major PA form present, with concentrations of lycopsamine only 1 µg/kg and no other PAs detected. These studies have tended to focus on the floral source of *Echium vulgare,* a plant that may be a targeted nectar source for monofloral honey or incidental in multifloral honey mainly in the South Island of New Zealand, but is not representative of all New Zealand honey varieties or production regions. Consequently, there is not a robust national dataset, which is needed to support risk assessment for honey consumers in New Zealand. Further, as the apiary environment in New Zealand differs from the profile overseas, including in Australia, and also region-to-region in New Zealand, the risk profile from PAs could be expected to not be comparable to overseas assessments. To address data gaps in the New Zealand risk profile, a program of monitoring New Zealand honey has been undertaken over several years to build a comprehensive dataset on the concentration, distribution, and fingerprint of common PAs and their N-oxides. The core 18 PAs and N-oxides tested were echimidine, echimidine-N-oxide, intermedine, intermedine-N-oxide, indicine, lasiocarpine, lasiocarpine-N-oxide, lycopsamine, lycopsamine-N-oxide, retrorsine, retrorsine-N-oxide, senecionine, senecionine-N-oxide, seneciphylline, seneciphylline-N-oxide, senecivernine, senecivernine-N-oxide, and senkirkine. Other PAs, such as echivulgarine and riddelliine, were also additions to the testing in individual years. This program undertook to analyze honey from drums, as aggregates of the PA content in hives and apiaries, and tanks, where honey is collated and blended to maintain product consistency before batching into retail packs. Specific aims from this research have been to characterize the magnitude and risk of lifetime exposure of honey consumers to PAs, and identify any opportunities for risk management of high concentrations or exposures though understanding the causes of variation in PA levels and profiles. 

## 2. Results

### 2.1. Occurrence of Pyrrolizidine Alkaloids in New Zealand Honey

Over the five summer seasons in which honey was sampled, a total of 776 drum samples, and 274 samples from tanks wherein the honey was batched and destined for retail, were analyzed for PAs (Table 1). Overall, lycopsamine is the most common PA across New Zealand honey. 

**Figure 2:**
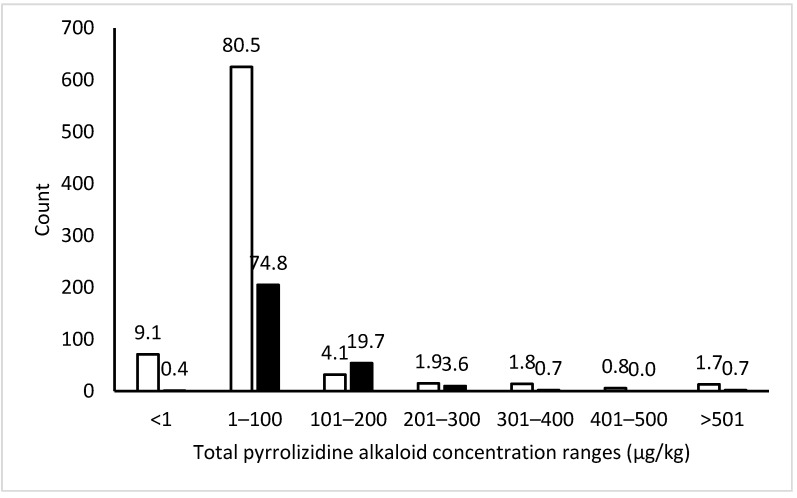
Distribution of total pyrrolizidine alkaloids concentrations in 776 samples of drum honey (white), and 274 samples of tank (retail) honey (black), with percentage recorded in data labels.

The overall total PA profile in honey shows the majority of drum and tank (retail) samples are under 100 µg/kg, with a long tail in total PA concentrations out to a maximum of 2777 µg/kg (Figure 2). For ease of comparing the PA fingerprints between the five surveys, as well as the regions and seasons, PA levels were categorized as low (<20 µg/kg), moderate (20–150 µg/kg), and high (>150 µg/kg).

#### 2.1.1. 2013/2014. Survey

Lycopsamine was the most commonly reported PA, being present in 103 of the 122 samples (84%), and at concentrations of >20 µg/kg in 23% of the samples, with five samples exceeding 150 µg/kg (Table S1). Echimidine and retrorsine were also both commonly reported (23% and 22% respectively), although only a single sample had both PAs present. Both of these PAs also had higher concentrations, with 6–7% of samples with >20 µg/kg, and 2% of samples exceeding 150 µg/kg. Senecionine and seneciphylline were most common, although not exclusively, in those honeys containing retrorsine, with an overall rate of reporting in 16% of the honeys tested. However, only one sample for each of senecionine and seneciphylline exceeded 20 µg/kg. Senkirkine was reported in three samples, all at levels below 20 µg/kg.

#### 2.1.2. 2016/2017. Survey

Lycopsamine was the most commonly reported PA, being present consistently through both harvests (Table S2). Retrorsine also appeared in both harvests, with two drums in each harvest having concentrations of >20 µg/kg. Senecionine and seneciphylline were both only present in the second harvest drums, the latter detected in all 19 drums tested. Senkirkine was also only present in the second harvest, although at low levels (<10 µg/kg) in a third of the drum samples. No echimidine, echivulgarine, or N-oxides for any of the PA congeners were detected in any sample.

#### 2.1.3. 2017/2018. Survey

Of the four regions samples were collected from, Northland honeys had the highest PA concentrations, averaging a total PA concentration of 82 µg/kg (Table S3). However, this was driven by the findings in the second harvest (mean: 144 µg/kg) where 31% of samples exceeded 150 µg/kg, compared to only a single sample in the first harvest (mean: 15 µg/kg). For all of the higher (>150 µg/kg) Northland honey results, retrorsine was the main contributing PA, with its higher prevalence in the second harvest leading to the overall higher concentration here. Senecionine and seneciphylline were also predominant in the second harvest samples, but present in only 4/50 samples in the first harvest. The sum of intermedine, lycopsamine, and indicine was generally consistent through both harvests at similar levels, and, in the first harvest, reflected the main contributing PA in most of the samples.

Contrasting with Northland, the mean total PA content was more consistent between the two harvests for Hawkes Bay honey (1st harvest: 17 µg/kg; 2nd harvest: 30 µg/kg), resulting in an average of 22 µg/kg. Although the second harvest had a slightly higher upper range than the first, none of the Hawkes Bay honey samples exceeded 150 µg/kg. As with the Northland honeys, retrorsine contributed to a number of the higher PA results, although there was more consistency in occurrence between the two harvests. The sum of intermedine and lycopsamine was the other main PA type seen consistently in the samples between seasons. East Cape honey also averaged 22 µg/kg, with only a single sample exceeding 150 µg/kg. Similarly, the Wairarapa honeys had a single sample exceeding 150 µg/kg, and a mean total PA content of 43 µg/kg. East Cape honeys were almost exclusively contributed to by retrorsine, with lycopsamine being only minor, and seneciphylline only present in three samples. Lastly, the sum of intermedine, lycopsamine, indicine, and their N-oxides, were consistent in Wairarapa honey. Retrorsine and seneciphylline-N-oxide were only seen in single samples. Echimidine, lasiocarpine, and senkirkine were only rarely detected across any of the samples taken in this survey.

#### 2.1.4. 2018/2019. Survey

As with the previous surveys, the most common PA grouping detected in the 2018/2019 survey was intermedine and lycopsamine (and their N-oxides), being present in 72% of samples, and at higher concentrations (>20 µg/kg) in 17% (Table S4). Seven samples had intermedine, lycopsamine, indicine, and their N-oxides exceeding 150 µg/kg, with individual high results of 1211 and 2253 µg/kg seen in two samples. 

The grouping of senecionine, seneciphylline, senecivernine, and senkirkine (and their N-oxides) was the second most prevalent in the samples, being reported in 35%, and at higher concentrations in 8% of samples. However, only four samples exceed 150 µg/kg for this grouping.

Echimidine was reported in 21% of samples, and also in a number of samples with higher concentrations, with 7% of all samples having echimidine reported at >20 µg/kg and 3% >150 µg/kg.

Retrorsine was reported in 20% of samples, and at >20 µg/kg in 4% of samples, with two individual high results of 1069 and 1304 µg/kg. As with the 2013/2014 survey, there was little crossover of retrorsine and echimidine in samples, with only eight samples being positive for both, and in all these cases, the concentrations of either PA were below 20 µg/kg. Riddelliine was analyzed for in 191 (57%) of the samples with no detections reported, and lasiocarpine and its N-oxide were also absent in all samples.

#### 2.1.5. 2019/2020. Survey

The sum of intermedine, lycopsamine, indicine, and their N-oxides were the key PAs across most of the samples, being present in all but one sample across the four honey packers, and, with a few exceptions, driving the majority of higher results (Table S5). Echimidine and its N-oxide was also highly prevalent, and was the major PA in a smaller number of the higher results. Contrasting with the 2017/2018 and 2018/2019 surveys, was that retrorsine was only a minor contributor to PA levels, with concentrations of retrorsine being intermittent across samples and only at low levels. Senecionine, senecivernine, and seneciphylline were also present in a small proportion of honeys and at similarly low levels (<24 µg/kg). Riddelliine was detected in six of the samples, but at concentrations of only 1–3 µg/kg, whereas senkirkine was similarly as rare (three positive samples), and at levels of 1–2 µg/kg. No lasiocarpine was detected in any sample.

### 2.2. Spatial Variation in Pyrrolizidine Alkaloids in Honey

**Figure 3:**
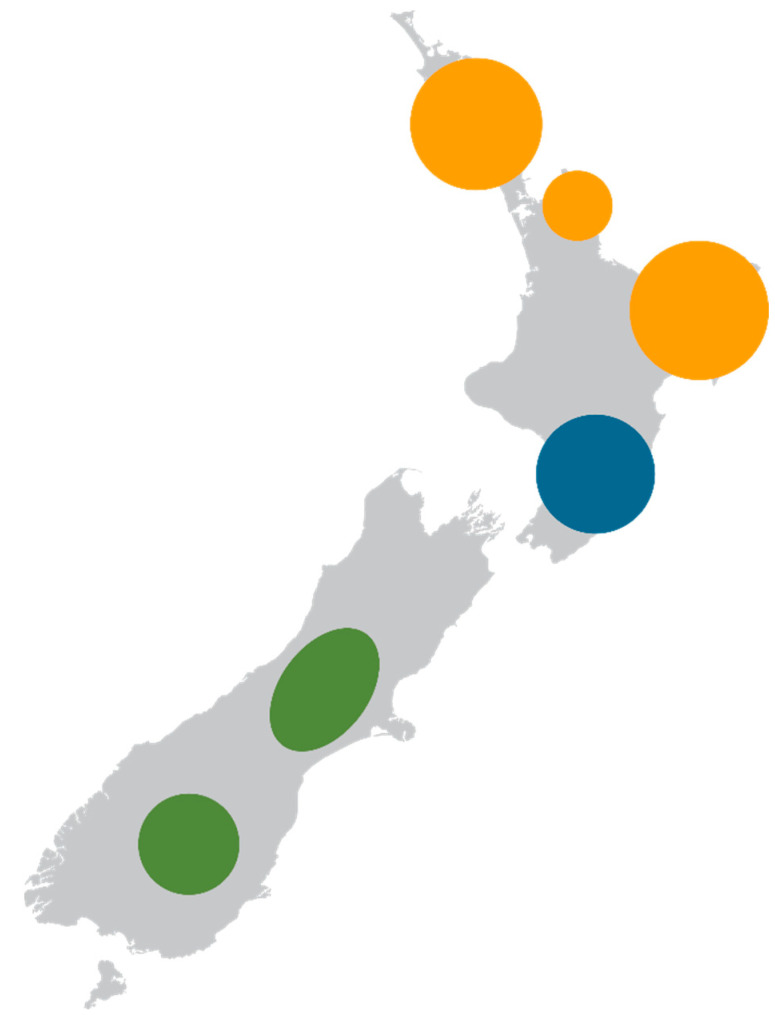
Regional prevalence of PA types in New Zealand honey (retrorsine and senecionine-type pyrrolizidine alkaloids shown in orange; lycopsamine-type pyrrolizidine alkaloids shown in blue; echimidine shown in green), circles scaled to fit the region size.

To understand spatial variation in the five New Zealand honey PA surveys, samples were traced back to apiary sites. GIS mapping of samples identified a higher prevalence of certain PA types in some regions (Figure 3). Retrorsine and senecionine PA types had a higher occurrence in Northland, Coromandel Peninsula, and the East Cape regions of the upper North Island of New Zealand. Lycopsamine PA types had a higher occurrence in the Wairarapa and Manawatu areas of the lower North Island. Echimidine PA types had a higher occurrence in the central high country and Central Otago areas of the South Island. 

### 2.3. Botanical Origin of Pyrrolizidine Alkaloids in Honey

The likely botanical origins of the reported PA types in New Zealand honey were assessed using three botanical reference databases to identify species from known PA-containing families prevalent in New Zealand (Table 2) [18,19,20,21,22].

In New Zealand, the flowers of two endemic species of *Apocynaceae* were also analyzed to establish if they were potential botanical origins of PAs: *Parsonsia capsularis* (NZ Jasmine) and *Parsonsia heterophylla* (Kaihua) (Table 3). 

### 2.4. Exposure Assessment 

As the dataset most reflective of the final consumed product by New Zealanders, the results of the 2019/2020 retail honey survey were assessed for a simulated lifetime exposure. The exposure assessment was conducted using a lifetime probabilistic dietary exposure model that accounted for market share (Table 4). A number of national and international agencies have reviewed the toxicological datasets for pyrrolizidine alkaloids over the last 20 years, deriving a broad range of toxicological health-based guidance values or points of departure for carcinogenicity assessment [2,3,23,24,25]. The contemporary Joint FAO/WHO Expert Committee on Food Additives (JECFA) 2020 review concluded that the genotoxic mode of action of PAs does not allow derivation of a health-based guidance value for chronic toxicity [3]. JECFA, however, selected a benchmark dose level (BMDL_10_) for liver hemangiosarcoma in female rats treated with riddelliine. The lowest BMDL_10_ value for riddelliine, derived with the two-stage model, was 182 μg/kg bw/day, or 0.182 mg/kg bw/day. To enable risk characterization of exposures to PAs through New Zealand honey, the JECFA BMDL_10_ value was adopted as the point of departure. Mean, median, and 95th percentile exposure assessment results were characterized using a margin of exposure (MOE) approach:

Margin of exposure = Point of departure/Exposure(1)

On an individual year of life basis, the dietary exposure range was not moderated to long-term patterns of consumption, and had a larger range (Table 5).

## 3. Discussion

### 3.1. Occurrence of Pyrrolizidine Alkaloids in Honey

The New Zealand survey results outline a pattern of general PA contamination of honey, largely with levels below 100 µg/kg total PAs, however, with a long tail out to a maximum reported value in drum honey of 2277 µg/kg (Table 2, Figure 2). Notably, compared to the surveys of honey from drums, the honey from retail batches contained a tighter distribution, with considerably fewer non-detect results and highly elevated results. This finding is likely a consequence of the blending that occurs of multiple drums to produce a retail tank, moderating very high PA results with honey containing low or no PAs. A similar difference between bulk and retail honey datasets had also been reported in Germany and Latin America [2,26].

The New Zealand survey findings are largely comparable to the reporting of PAs in honey from overseas, for example, falling well within the compiled international range of 0.3–5600 µg/kg (*n* = 19,698) [3]. Median concentration across the New Zealand raw honeys surveyed of 7–12 µg/kg are comparable to those reported in Chile, Guatemala, Italy, Mexico, and Spain [25]. As the PA profile in honey is contingent on the flora growing around apiaries, it can differ between countries. Findings of lycopsamine and echimidine as the predominant PA types have been reported in retail honey purchased in Australia, Chile, and China [10,26,27].

The identification of seasonal and inter-year variation in the New Zealand surveys aligns with the findings of other studies. For example, in Germany, there was a major increase in mean total PAs in honey going from spring to summer 2016, whereas mean results for summer 2016 were also close to 10-fold greater than those of the preceding year’s summer [28]. Notably, in this study of German honey, there was also a changing PA profile between seasons, with lycopsamine- and echimidine-type PAs a greater contributor to overall levels found in spring, but largely minimal compared to the senecionine-type PA reported in summer that was linked to flowering of *Jacobea vulgaris.* This supports the finding of seasonality for retrorsine and senecionine in the New Zealand honeys from Northland, Coromandel Peninsula, and East Cape.

Predominantly the parent PAs were more commonly reported in honey across the five survey years, and at higher concentrations than their corresponding PA-N-oxides. Some exceptions were noted for echimidine and lycopsamine, including in the sample reporting the highest result of 2277 µg/kg, where the ratio of lycopsamine to its N-oxide was 1:6, with the latter reporting a concentration of 1952 µg/kg. Prior research has reported that processing and storing honey appears to reduce the PA-N-oxides content, often very rapidly [16,29,30]. However, research findings show this does not equate to conversion to the parent PA, with levels of various parent PAs remaining stable during storage [29,30]. Conversion to a masked form that is not reported during analysis may underestimate the true concentration that presents a dietary risk. Conversely, as all the honey samples analyzed in the New Zealand surveys were pre-retail, further loss of PA-N-oxide during storage to a form that is low toxicity would result in an overestimate of the exposure for consumers.

### 3.2. Botanical Profiles

Considering the prevalence of species for PA-containing families in New Zealand, a floral origin of echimidine from *Echium* spp. aligns well with the previous analysis of New Zealand *Echium vulgare* honeys (Table 2) [16,17]. Of the 117 honey samples taken from apiaries in the Canterbury, Otago, and Southland regions of New Zealand in the 2013/2014 and 2018/2019 surveys, 74 had reported concentrations of echimidine at an average concentration of 72.3 µg/kg (range: 1–528 µg/kg). *Echium vulgare* is widely distributed in these regions. In contrast, echimidine was rare in North Island and Tasman/West Coast honeys in the 2013/2014 and 2018/2019 surveys, with only 28 out of 378 samples with a quantified concentration, at an average of 6 µg/kg (range 1–75 µg/kg).

Although New Zealand has an extensive endemic Asteraceae flora, many of the species are not prevalent in the habitats where apiaries are located. *Brachyglottis repanda* is an exception to this, and occurs frequently in bush and scrubland. 

Analysis of the flowers of both *Parsonsia capsularis* and *Parsonsia heterophylla* established they contain high levels of lycopsamine-type PAs, but negligible or no detected concentrations of retrorsine-, senecionine-, or echimidine-type PAs (Table 3). Consequently, both species may be botanical contributors to lycopsamine-type PAs in honey, particularly as they are well represented in bush margins where apiaries may be sited. The presence of lycopsamine-type PAs in New Zealand *Parsonsia* spp. aligns with the results of a recent study that identified lycopsamine and its N-oxide as the predominant PAs in flowers, nectar, and pollen of *Parsonsia straminea,* concluding that this species was a likely botanical origin for these PAs in honey [10]. Lycopsamine-type PAs were also identified in other *Parsonsia* spp., as well as more broadly across the Apocynaceae [31].

### 3.3. Linking Regional and Botanical Profiles and Seasonality

Regional prevalence of PA types was able to be linked to specific botanical profiles either through identification of suitable habitats or land management practices, or seasonality of harvest, such as late season (Table 6). The seasonality variation may be related to the greater incidence of flowering weed species later in summer.

The identification of risk factors at a regional level for PA contamination of honey is invaluable to support the development of risk communication and risk management tools to limit or mitigate this risk. A developed plant pack resource for apiarists, both commercial and hobbyist, provides a visual resource to identify key flowering plant species and habitats that are risk factors for PA contamination, allowing apiarists to take action to manage the risk [32]. The guidance takes a precautionary approach to identifying species related to PA-containing plants as being of potential concern (e.g., *Phacelia tanacetifolia*, Phacelia, purple tansy) even in the absence of reported PA occurrence.

### 3.4. Risk Characterization

In New Zealand, risk management for genotoxic carcinogens aims for an MOE of 10,000 or higher to identify if the exposure is of low concern for public health [33]. The lifetime exposure assessment and risk characterization of the PA content in New Zealand retail honey supports the risk being within the current tolerance for a dietary carcinogen (MOE > 10,000; Table 4). None of the percentiles of the modelled lifetime exposure exceeded 6 ng/kg bw/day, or, when considered against the health characterization value, fell below an MOE of 30,000. 

When considered on an individual year basis, the range of exposure is much greater than that averaged over the lifetime, and for younger age groups, a proportion of the population would have an exposure value leading to an MOE of less than 10,000, for example, the top 14% in the case of 5-year old children (Table 5). Given the low proportion of high PA-containing honeys established in the market study, a sustained level of exposure to these is unlikely over a lifetime, and as intake to body weight ratios reduce with maturity, the exposure drops, hence, a notable dietary risk is unlikely. It is also uncertain how relevant these periods of elevated exposure are for considering against a toxicological point of departure designed for hazard characterization of lifetime exposure. However, where some beekeepers and their families may be high consumers of their own honey, which may, in some circumstances, have much higher PA levels, they may receive a higher consistent level of exposure, and, potentially, a chronic health concern. Such a finding was reported in a study of German honey, with the risk assessment concluding average adult and child consumers had MOEs > 10,000 with normal and high PA contamination honeys [28]. However, the authors considered exposure to be a risk for beekeepers, their families, and regular highly brand-loyal customers. In New Zealand, the ongoing efforts to educate apiarists on the risk factors for PA contamination of honey will likely reduce any exposure risks from higher PA levels [32].

The estimated exposures to PAs in New Zealand are comparable with recent overseas estimates for honey. JECFA estimated an exposure of 0.02 and 3.9 ng/kg bw/day (lowest lower bound (LLB)– highest upper bound (HUB)) for the mean consumers of honey, and between 5 and 26 ng/kg bw/day (LLB–HUB) among the high consumers in the adult population [3]. Similarly, in an estimate for the European adult population, the mean chronic exposure via the consumption of honey ranged between 0.1 and 7.4 ng/kg bw/day (minimum LB–maximum UB), whereas for high consumers, it was between 0.4 and 18 ng/kg bw/day (minimum LB–maximum UB) [2].

### 3.5. Uncertainty

Within the risk assessment, a number of decisions were required to address known data gaps, and these all introduce some uncertainty into the conclusion. Table 7 outlines the actions that have been undertaken, and the magnitude of the uncertainty it could introduce into the conclusions. 

The major identified uncertainty related to assumptions on an equivalent potency of different PAs when using a total PA value for the exposure assessment. Two studies have considered the relative potency of different PAs. Merz and colleagues proposed interim relative potency (REP) factors for a number of abundant PAs, based on the concept that all carcinogenic, genotoxic PAs share a common mode of action, i.e., the metabolic formation of reactive dihydropyrrolizine (DHP) and/or reactive DHP esters which attack cellular nucleophiles [35]. Xia and colleagues concluded that retrorsine formed the highest amount of DHP-deoxyguanosine- and -adenosine-adducts, followed by lasiocarpine, and with lycopsamine the least potent of those looked at [36]. Furthermore, lycopsamine produced less than one percent of the amount of adducts produced by retrorsine, and riddelliine-N-oxide formed more adducts than senkirkine. 

This research is indicative that assuming equal carcinogenic potential for all PAs and adding their cumulative effect with respect to their carcinogenicity will likely overestimate the risk. However, currently available data are not sufficient to identify relative potency factors for all PAs commonly in the diet. This is supported by the JECFA conclusion that the current available data were not sufficient to identify relative potency factors for different 1,2-unsaturated PAs in order to evaluate the possible effects of combined exposure [3]. 

As further research is undertaken on pyrrolizidine alkaloids, both internationally and within New Zealand, there will be the opportunity to refine the decisions made in the assessment model, and improve the robustness of the assessment and the resulting conclusions.

Understanding other dietary contributors in New Zealand will also be key to placing the exposure through honey in context, and assessing the overall dietary intake. For example, JECFA estimated the mean and high-percentile chronic dietary exposures to PAs through adult consumption of tea range from 1 to 130 ng/kg bw/day and 10 to 260 ng/kg bw/day, respectively [3]. A similar pattern of exposure in New Zealand would imply teas were a comparable or greater source of exposure to PAs than honey. A study to characterize and exposure assess PA concentrations in teas, herbals teas, and infusions available in retail in New Zealand is currently being conducted. Further research on other known contributors, for example, herbs, would also be beneficial to refine exposure estimates.

## 4. Conclusions

Pyrrolizidine alkaloids are important botanical toxins in the diet due to concerns over their carcinogenicity from chronic exposure. PAs are transferred into honey through bees foraging flowers of PA-containing plants, with the consequence that honey may be an appreciable contributor to dietary exposure. To characterize the prevalence of PAs in New Zealand honey, a multiple year program of testing drum and tank honey samples was conducted. PAs were detected in ~90% of drum honey samples, and in nearly all tank samples, with the mean total PA concentration ranging from 16–74 µg/kg. Geographic variation was evident in the fingerprint of PAs, which was concluded to result from the occurrence of different PA-containing plant species in the apiary environments of each region. Characterizing the key PA-containing plant species and their habitats has enabled the development of educational material for apiarists on risk factors for PA contamination of honey. 

Assessment of lifetime exposures from the retail honey survey dataset identifies that there is not an immediate cause for concern for general consumers with the prevalence of PAs in New Zealand honey. Uncertainties over high consumption habits, however, limit characterizing this risk for sensitive populations. General New Zealand exposures are also comparable to overseas estimates through honey, although identification of other dietary contributors will be important to complete the risk assessment for PAs. 

## 5. Materials and Methods

### 5.1. Sample Collection

Sample collection of New Zealand honeys was undertaken over five summer seasons. Drum honey samples were collected in 2013/2014 (122 samples), 2016/2017 (65 samples) 2017/2018 (255 samples), and 2018/2019 (334 samples). Samples of 50 mL were collected by producers and apiarists from honeys extracted into 30 kg drums. A drum may be a composite of raw honey from several hives within a region. 

The 2013/2014 and 2018/2019 surveys had a national distribution; whereas the 2016/2017 survey focused solely on the Northland region; and that in 2017/2018, on four North Island regions (Northland: 101 samples; Hawkes Bay: 78 Samples; East Cape: 60 samples; Wairarapa: 16 Samples). The 2016/2017 and 2017/2018 surveys were able to take from the early summer and late summer harvests in some of the regions (Northland and Hawkes Bay).

In the 2019/2020 survey, 274 samples of retail batches of honey were taken from across four retail producers (producer 1: 60 samples; producer 2: 134 samples; producer 3: 51 samples; producer 4: 29 samples). The 50 mL samples were taken from tanks representative of batches of honey destined for retail, totaling 924 tonnes, representing approximately three quarters of the 1200 tonnes of honey in New Zealand retail over a six-month period. Weights of tanks were varied, but were on average 3000 kg. 

Flowers of *Parsonsia capsularis* and *Parsonsia heterophylla* were collected from bush margins in the Bay of Plenty in the Southern Hemisphere summer of 2020. Samples were sent to Manaaki Whenua—Landcare Research for botanical identification. 

### 5.2. Analytical Testing

Analytical testing for PAs in New Zealand honey was undertaken by two laboratories in New Zealand: AsureQuality, Auckland; and Analytica Laboratories, Hamilton. Analysis generally focused on a core group of 18 congeners (10 PAs and 8 PA-N-oxides), but had some differences across the five surveys, as the targeted suite of relevance to New Zealand honey was refined (Table 8). Structural formulae of these analyzed PAs and N-oxides are presented in the recent JECFA safety evaluation [3]. Further in the 2013/2014 survey, the PAs acetyl-echimidine, acetyl-vulgarine, echiumine, echiuplatine, vulgarine, and all of their respective N-oxides were also analyzed. 

For the honeys, samples were analyzed by both laboratories by the method of Betteridge and colleagues [16]. Essentially, honey is extracted with dilute sulfuric acid, and applied to a pre-conditioned strong cation exchange resin. The resin is washed, and PAs are eluted using methanolic ammonia solution, which is evaporated and reconstituted for analysis by UPLC-MS/MS. A Waters Acquity HSS T3 column is used to separate analytes, with MRM monitoring by a Sciex 6500 triple-quad mass spectrometer using an electrospray ion source. AsureQuality used an Agilent 1290 Infinity HPLC system interfaced with a Sciex TRIPLE QUAD 6500 mass spectrometer. Analytical standards for the PAs were supplied by PhytoLab GmbH & Co. KG, Vestenbergsgreuth, Germany.

Sampled flowers of *Parsonsia capsularis* and *Parsonsia heterophylla* were received fresh by Analytica Laboratory and immediately frozen at −20 °C. They were ground to a fine powder in a mortar and pestle, after freezing with liquid nitrogen. A 50 mg portion was extracted using dilute sulfuric acid, and processed in the same manner as for honey, with additional dilution prior to chromatography as required to allow for the higher concentration range. A dry matter was obtained for each sample, and the results expressed as mg/kg dry matter. 

The analytical limits of reporting (LOR) were 1–3 µg/kg across the analyzed congeners. A number of the PAs analyzed are typically absent or minimal in New Zealand honey, for example, riddelliine and lasiocarpine. Contrasting with this, some of the PA congeners may be typical for a honey, but in an individual sample, at a level below the LOR. As a consequence, it was considered that an approach of substituting left-censored data (<LOR) with a positive value in deriving a total PA content for a sample would likely overestimate true levels, and underemphasize the importance of the PA congeners detected. A lower bound approach, whereby results below the LOR were replaced with “zero”, was chosen to reduce this uncertainty. Given very few samples were fully left-censored, it was considered this would have negligible risk of underestimating exposures.

### 5.3. Exposure Assessment Model

The assessment of the chronic exposure nature of pyrrolizidine alkaloids through honey presented a range of challenges that required tailoring a specific probabilistic lifetime exposure model. Exposure was considered over a lifetime basis, thus, allowing consideration of the impact of the probabilities year to year of consuming different amounts of honey, accounting for the specific distribution in the recorded PA concentrations of retail honey.

(2) $$D_{L}=\left(\sum_{i=0}^{70}D_{i}\right)÷70,$$

(3) $$D_{i}=\sum_{n=1}^{XJi}D_{n}=\left(D_{n}+D_{n+1}\cdots +D_{XJi}\right)÷BW_{i},$$

where *D_L_* is the average annual exposure over a lifetime (µg/kg bw), *D_i_* is the annual exposure for a specific year of life between 0–70 years (µg/kg bw). *D_n_* is the exposure from the instances in a year of life from an index of between 1 and a random whole number within the upper limit for that year of life (*XJi*), each instance equal to I × XC. Where I is the honey intake from one jar of 0.5 kg, XC is a cumulative probability-derived total PA concentration, simulating market share from the 2019/2020 PA dataset (µg/kg). *BW_i_* is the body weight for that year of life (kg).

The 2019/2020 survey data was selected for the occurrence data for the exposure assessment. As this survey analyzed tanks of honey destined for batching as retail products in New Zealand, it was the dataset most reflective of the final consumed product. The other surveys sampled from drums, which may be further blended to derive a consistent product, as well as potentially being bound for export, would not reflect as well the commercial product that is consumed in New Zealand. To reflect the market share data in the data, the tonnage of each batch was divided by the total tonnage (924 tonnes) for all the batches in the dataset to arrive at a proportion of the market share. Sorting on an ascending basis by total PA concentration, the cumulative probability for a specific PA concentration based off the market share of each batch was calculated. A representative dataset of the PA concentrations in retail honey of 185,000 cells was then simulated from this cumulative probability, based on 10% of the total 500 g jars entering retail annually (in practice, reflecting 10% of the product accounted for in the batches tested in the 2019/2020 survey once adjusted to an annual production volume). 

A key requirement of a dietary exposure model is the use of representative intake data for that food or food group. The mean consumer daily consumption amount for honey from the Ministry of Health 2008/2009 Adult Nutrition Survey and 2002 Child Nutrition survey are used in the exposure assessment [37,38]. These are 8.18 g/day for adults 18+ years, and 5.39 g/day for children 5–14 years. An individual jar of honey (approximately 80% of which in New Zealand retail are 500 g) will provide a fixed source of exposure to a PA concentration over a number of daily portions until the jar is empty. Consequently, the mean daily consumption amount of honey was extrapolated to an annual value and then divided on a 500 g jar basis to provide the upper range of jars consumed per year by a frequent honey consumer. This was calculated at four jars per year for children, and six per year for adults.

**Figure 4:**
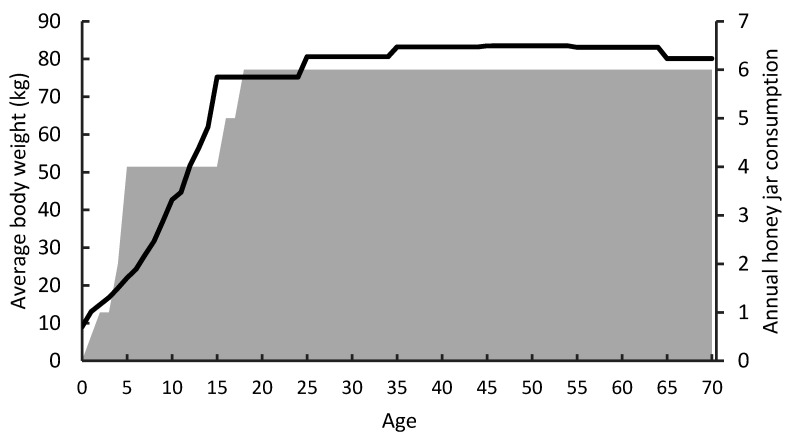
Average bodyweights (black line), and estimated range of annual honey 500 g jars consumed (grey area block) for each age (0–70 years) for a lifetime exposure model of pyrrolizidine alkaloids in honey [36,37].

Using Microsoft Excel 365, the exposure to PAs in each year of an individual’s life from 0–70 years of age was modelled, with the body weight for each year of life based on those reported in the New Zealand Child Nutrition Survey and Adult Nutrition Survey as average for that age or age range (Figure 4) [37,38]. For each iteration in the model, annual honey consumption was randomly assigned within the range of estimated annual jar consumption (Figure 4). Values are based upon the estimated range of 1–4 jars per year for children (5–14 years of age), and 1–6 per year for adults (18–70 years), with an extrapolated gradient between them for toddlers and older teenagers. New Zealand recommendations are that infants should not consume honey, so a zero-consumption amount (0 g) was listed for age 0–1 years [39]. Each instance of honey consumption was combined with a random total PA concentration (converted to the PA content of the 500 g jar) from the market share adjusted 2019/2020 retail survey dataset. For each iteration, the exposures were converted to a body weight basis for that specific year of life. 

For each year between 0–70 years of age, 100,000 iterations were run in parallel, and the findings averaged on a lifetime basis. Annual exposures for individual years of age (5 years and 15 years), as well as for average male and female adults (18+ years), were also extracted to understand ranges of exposure. To enable comparison to the toxicological point of departure, all exposures were converted into a daily value and reported as mean, median, and 95th percentiles of the model outputs.

(4) $$D_{i}=3.71=\left(\left(\;0.5\times 104\right)+\left(0.5\times 22.7\right)+\left(0.5\times 12.5\right)+\left(0.5\times 31.6\right)\right)÷23,$$

an example calculation of *D_i_* for an age of 5 years, where *XJi* = 4, derives an annual intake of 3.71 µg/kg bw. When converted to a daily basis to compare with the toxicological point of departure, this is 10.2 ng/kg bw/day, or an MOE of 17,800.

## Figures and Tables

**Table 1:** Overview and descriptive statistics for the five New Zealand honey pyrrolizidine alkaloid (PA) surveys.

Survey Year	Honey Type	Sample Number	Samples < LOR Total PA (%) ^1^	Total PA Concentration (µg/kg)					Predominant PA Types ^2^
				5th% Ile	Median	Mean	95th% Ile	Maximum	
2013/2014	Drum	122	12 (10)	0	12	62	350	810	L > E > R > S
2016/2017	Drum	65	2 (3)	1	7	16	63	130	L > R > S
2017/2018	Drum	255	16 (6)	0	12	47	219	641	L > R > S
2018/2019	Drum	339	43 (13)	0	12	56	244	2277	L > S > E > R
2019/2020	Tank	274	1 (0)	8	46	74	199	912	L > E > R/S

^1^ LOR = limit of reporting. ^2^ R = retrorsine and its N-oxide; S = senecionine, senecivernine, seneciphylline, and their N-oxides; L = intermedine, lycopsamine, indicine, and their N-oxides; E: echimidine and its N-oxide.

**Table 2:** PA plant families and species with prevalence in New Zealand (common name in brackets) [18,19,20,21,22].

Plant Family	PA Type	Species in New Zealand
Asteraceae	L	*Eupatorium cannabinum* (Hemp-agrimony)
	R/S	*Brachyglottis repanda* (Rangiora) *Erechtites hieraciifolia* (American fireweed) *Jacobaea vulgaris* (Ragwort) *Senecio bipinnatisectus* (Australian fireweed) *Senecio biserratus* (Fireweed) * Senecio skirrhodon (Gravel groundsel) ^#^ *Senecio vulgaris* (Common groundsel)
Boraginacaea	E	*Echium plantagineum* (Patterson’s curse) *Echium vulgare* (Viper’s bugloss)
	L	Amsinckia calycina (Yellow gromwell) ^†^ Cynoglossum amabile (Chinese forget-me-not) Borago officianalis (Common borage) Myosotis arvensis (Field forget-me-not) Symphytum officinale (Comfrey) Symphytum x uplandicum (Nyman Russian comfrey)

R = retrorsine and its N-oxide; S = senecionine, senecivernine, seneciphylline, and their N-oxides; L = intermedine, lycopsamine, indicine, and their N-oxides; E: echimidine and its N-oxide. * Syn *Senecio flaccidus*. ^#^ Syn *Senecio madagascariensis.* ^†^ Syn *Amsinckia angustifolia, Amsinckia hispida.*

**Table 3:** Pyrrolizidine alkaloid content of flower heads of *Parsonsia heterophylla* and *Parsonsia capsularis*.

Pyrrolizidine Alkaloid	Pyrrolizidine Alkaloid Concentration in Apocynaceae Species Flower Heads (mg/kg dw)	
	*Parsonsia heterophylla*	*Parsonsia capsularis*
Intermedine	22	310
Intermedine N-oxide	3600	59,000
Sum of Lycopsamine and Indicine	1000	<1
Lycopsamine N-oxide	51,000	520
Senecionine	0.044	<1

**Table 4:** Dietary exposure modelling for an averaged lifetime exposure and risk characterization for pyrrolizidine alkaloids in New Zealand tank (retail) honey using a margin of exposure (MOE) against the JECFA BMDL_10_ for riddelliine (182 µg/kg/bw/day) [3].

Survey	Mean Exposure (ng/kg bw/day)	Mean Exposure (MOE)	Median Exposure (ng/kg bw/day)	Median Exposure (MOE)	95th Percentile Exposure (ng/kg bw/day)	95th Percentile (MOE)	Percentile of Exposure at Which MOE < 10,000
2019/2020	4.6	39,400	4.6	39,600	5.4	33,200	n/a

**Table 5:** Dietary exposure modelling for a single year of life exposure at different ages and risk characterization for pyrrolizidine alkaloids in New Zealand tank (retail) honey using a margin of exposure (MOE) against the JECFA BMDL_10_ for riddelliine (182 µg/kg/bw/day) [3].

Age/Gender (Years Old)	Average Bodyweight (kg)	Mean Exposure (ng/kg bw/day)	Median Exposure (ng/kg bw/day)	95th Percentile Exposure (ng/kg bw/day)	Percentile of Exposure at Which MOE < 10,000
5	23	11	8.5	26	86th
15	54	4.6	3.7	11	99th
Female (18+)	70	4.9	4.2	12	99th
Male (18+)	82	4.2	3.7	10	n/a

**Table 6:** The linkage between prevalence of PA types in regions and PA species due to favorable habitats.

PA Type	Region	Habitat/Practice	Seasonality
Echimidine	South Island high country and Central Otago	Wild fields and gardens of *Echium* species	Not applicable for these regions
Lycopsamine	Wairarapa and Manawatu (Lower North Island)	Bush margins that contain *Parsonsia* vines Wild or commercial sites, and gardens containing borage, comfrey, and other Boraginaceae and *Eupatorieae*	Not applicable for these regions
Retrorsine/Senecionine	Northland, Coromandel Peninsula and East Cape	Forestry blocks felled in past 5 years Burnt, cleared, or barren land Weed infested pasture	Second harvest samples had a higher frequency of higher levels of PAs

**Table 7:** Outline of risk assessment actions introducing uncertainty and the estimated magnitude.

Action	Uncertainty	Consequence
Use of total PA values in risk assessment.	Relative potency of the toxicity of different PAs.	Major: Overestimate of toxicity by potential 1000-fold.
Use of lower bound approach (ND = zero).	Assumes absence of PAs when not detected.	Minor: Few results are fully ND; NDs are most common for PAs with minimal profile in NZ honey, e.g., riddelliine.
Use of a suite of 18 PAs and N-oxide	Potential occurrence of other PA congeners in the samples. Masked PAs from the loss of PA-N-oxides during processing and storage.	Moderate: Exposure could be underestimated if other PAs were notable. However, the tested suite aligns with PA testing recommendations in overseas studies [34].
Modelling consumption practices based only on 500 g retail jars.	~20% of honey is retailed in 250 g jars, other sizes up to 1 kg also sold.	Minor: Other jar sizes are a smaller proportion of retail; analysis of 250 g vs. 500 g shows limited impact on exposure.
Modelling lifetime exposure based on daily honey consumption amounts.	Minimal information available on long-term honey consumption practice.	Moderate: Exposure could be under/over-estimated in the population. Very high honey consumers (e.g., beekeepers) difficult to capture.
Weighting retail honey survey dataset based on market share.	10% of retailed honey not captured. Does not account for brand/honey type loyalty. Assumes nationwide distribution of all honey.	Moderate: Likely underestimates exposure for consumers with brand/type loyalty.
Consideration only of honey contribution to dietary PA exposure.	Presence of PAs, and dietary exposures to PAs from other foods available to NZ consumers is unknown.	Moderate: Risk characterization conclusion could be underestimated.

**Table 8:** Pyrrolizidine alkaloids analyzed in five surveys of New Zealand honey.

Pyrrolizidine Alkaloid	2013/2014	2016/2017	2017/2018	2018/2019	2019/2020
Echimidine	Tested	Tested	Tested	Tested	Tested
Echimidine-N-Oxide	Tested	Tested	Tested	Tested	Tested
Echivulgarine	Tested	Tested	-	-	-
Echivulgarine N-oxide	Tested	Tested	-	-	-
Intermedine	-	-	Tested ^1^	Tested ^1^	Tested ^1^
Intermedine-N-oxide	-	-	Tested ^2^	Tested ^2^	Tested ^2^
Indicine	-	-	Tested ^1^	Tested ^1^	Tested ^1^
Lasiocarpine	-	-	Tested	Tested	Tested
Lasiocarpine-N-oxide	-	-	Tested	Tested	Tested
Lycopsamine	Tested	Tested	Tested ^1^	Tested ^1^	Tested ^1^
Lycopsamine-N-oxide	Tested	-	Tested ^2^	Tested ^2^	Tested ^2^
Retrorsine	Tested	Tested	Tested	Tested	Tested
Retrorsine-N-oxide	Tested	Tested	Tested	Tested	Tested
Riddelliine	-	-	-	Tested ^3^	Tested
Senecionine	Tested	Tested	Tested ^4^	Tested ^4^	Tested ^4^
Senecionine-N-oxide	Tested	Tested	Tested ^5^	Tested ^5^	Tested ^5^
Seneciphylline	Tested	Tested	Tested	Tested	Tested
Seneciphylline-N-oxide	Tested	Tested	Tested	Tested	Tested
Senecivernine	-	-	Tested ^4^	Tested ^4^	Tested ^4^
Senecivernine-N-oxide	-	-	Tested ^5^	Tested ^5^	Tested ^5^
Senkirkine	Tested	Tested	Tested	Tested	Tested

^1^ In the 2017/2018, 2018/2019, and 2019/2020 surveys, testing of lycopsamine and intermedine are reported combined, with a proportion of results in 2017/2018 (46%), 2018/2019 (57%), and 2019/2020 (100%) also including indicine. ^2^ In the 2017/2018, 2018/2019, and 2019/2020 surveys, testing of lycopsamine-N-oxide and intermedine-N-oxide are reported combined. ^3^ In the 2018/2019 survey, riddelliine was analyzed in only a proportion of samples (57%). ^4^ In the 2017/2018, 2018/2019, and 2019/2020 surveys, testing of senecionine and senecivernine are reported combined. ^5^ In the 2017/2018, 2018/2019, and 2019/2020 surveys, testing of senecionine-N-oxide and senecivernine-N-oxide are reported combined.

## Data Availability

Data is contained within the supplementary material.

## Supplementary Materials

The following are available online at https://www.mdpi.com/article/10.3390/toxins13120843/s1. Table S1: Pyrrolizidine alkaloid concentrations in the 2013/2014 survey of New Zealand drum honey; Table S2: Pyrrolizidine alkaloid concentrations in the 2016/2017 survey of New Zealand drum honey; Table S3: Pyrrolizidine alkaloid concentrations in the 2017/2018 survey of New Zealand drum honey; Table S4: Pyrrolizidine alkaloid concentrations in the 2018/2019 survey of New Zealand drum honey; Table S5: Pyrrolizidine alkaloid concentrations in the 2019/2020 survey of New Zealand tank (retail) honey.
